# Causes of Raptor Admission to the Wildlife Rehabilitation Centre in Abruzzo (Central Italy) from 2005–2016

**DOI:** 10.3390/ani12151916

**Published:** 2022-07-27

**Authors:** Ciro Cococcetta, Thomas Coutant, Tommaso Collarile, Alessandro Vetere, Francesco Di Ianni, Minh Huynh

**Affiliations:** 1 Centro Veterinario Gregorio VII, Avian and Exotic Animals Department (AvianDoc), 52 Piazza di Villa Carpegna, 00165 Roma, Italy; cirocococ@gmail.com (C.C.); tommasocollarile@gmail.com (T.C.); 2 Service des Nouveaux Animaux de Compagnie, Centre Hospitalier Vétérinaire Frégis, 43 Avenue Aristide Briand, 94110 Arcueil, France; thomas_c_93@hotmail.fr (T.C.); timmean@hotmail.com (M.H.); 3 Department of Veterinary Science, Università Degli Studi di Parma, Strada Del Taglio 10, 43126 Parma, Italy; francesco.diianni@unipr.it

**Keywords:** raptor, birds of prey, cause of admission, wildlife rehabilitation center, Italy, mortality, morbidity

## Abstract

**Simple Summary:**

Nowadays, it is increasingly important to obtain data through the study of wild animals. There is no doubt that they provide a “summary” of the overall health of the natural environment. For years, wild animals that recovered at rescue centers have provided data on hazards in the wild, from those caused by human activities to more natural hazards such as viruses, bacteria, and parasites. Carcass analysis also provides data on potential environmental residues of pesticides and other toxic substances. The present retrospective study aims to provide an overview of the causes of admission to the Wildlife Rehabilitation Center; specifically of 2496 wild raptors from 2005 to 2016, in Abruzzo, central Italy. Results showed that the main causes of admission were trauma, nestling (including birds on their first flight attempt or presumed abandoned by their parents), and starvation.

**Abstract:**

The purpose of this retrospective study was to describe the causes of morbidity and mortality in free-ranging raptors admitted to a wildlife rehabilitation center (WRC) in Abruzzo Italy from 2005 to 2016 and the associated risk factors. A total of 2496 free-ranging raptors were included in the study. We analyzed the raptors’ medical records, epidemiological information, bird characteristics, cause of admission, final diagnosis, and outcome. The prevalence rates of nocturnal and diurnal raptors were 49% and 51%, respectively. Nocturnal raptors showed trauma as the primary cause of admission (45.8%, 558/1219), followed by nestling (including birds on their first flight attempt or presumed abandoned by their parents) (39.2%, 478/1219), and starvation (5.6%, 68/1219). Diurnal raptors showed trauma (73.1%, 934/1277), starvation (12.1%, 155/1277), and nestling (5.8%, 74/1277) accordingly. A description of the dangers for wild birds of prey in the Abruzzo region was provided to assist in the planning of rescue and rehabilitation activities in the WRC. Finally, the cause of admission, GAP, and BCS can be used as prognostic factors during the bird entry process.

## 1. Introduction

Birds of prey are considered valuable environmental indicators because of their role in the ecological food chain and because they are widespread across large geographical areas. Moreover, they are particularly sensitive to ecological changes [1,2]. As raptors are considered predators, they are believed to be useful as flagship species and to assess biodiversity. In fact, they are used as biodiversity indicator species and umbrella species, i.e., useful for protecting and studying other species that intersect with raptors both as a food chain and as reducing interspecies food competition [1,2].

In Europe, 36 species (64%) of the 56 raptors species have an unfavorable conservation status [3]. Forty species of diurnal, as well as ten species of nocturnal, birds of prey can be found in Italy, both breeding and migrating [4]. In particular, a region in south-central Italy, Abruzzo, was analyzed in the present study. That region hosts approximately 57.5% of the species surveyed in the country, including 17 diurnal and 6 nocturnal (Table 1).

The raptor species present in Italy are classified by the IUCN Europe Red List (International Union for Conservation of Nature) as Least Concern (LC), with the exception of the Red Kite (*Milvus milvus*) classified as NT Near Threatened, together with the Red-footed Falcon (*Falco vespertinus*) [5]. If the Italian IUCN Red List is consulted, a more complex scenario is observed, in which some species are found to be threatened with extinction. The species considered most at risk are the Griffon Vulture (*Gyps fulvus*), classified critically endangered (CR), and the Red-footed Falcon (*Falco vespertinus*), Montagu’s Harrier (*Circus pygargus*), the Western Marsh Harrier (*Circus aeruginosus*), Short-toed Snake Eagle (*Circaetus gallicus*), and the Red Kite (*Milvus milvus*), as vulnerable (VU). Those classified as near threatened (NT) are the Golden Eagle (*Aquila chrysaetos*) and the Black Kite (*Milvus migrants*). Two species of raptors are classified as not evaluated (NE), one nocturnal owl, the Short-eared Owl (*Asio flammeus*), and one diurnal raptor, the Merlin (*Falco columbarius*) [6]. All this highlights that many species appear to be worthy of greater protection and conservation at the national level.

Fragmentation of both breeding and hunting habitats, illegal killing, and contamination by pesticides and rodenticides (for all nocturnal raptors and, buzzards, and vultures, concerning the diurnal raptors) are the known risks in Italy for the raptors in the study [6]. Some species presented specific risks and threats, for example, the Short-toed Snake Eagle (*Circaetus gallicus*), a reduction of reptiles on which it feeds; Montagu’s Harrier (*Circus pygargus*), ground nesting for which agricultural mechanization at breeding sites may pose a threat, although of unquantifiable magnitude; Griffon Vulture (*Gyps fulvus*), as with most vultures, the species is threatened by both direct and indirect persecution (poisoned bites), but the main threat remains the reduction in food availability due to declining wild grazing and health regulations requiring carcass disposal; Barn Owl (*Tyto alba*), vehicle collisions and overhead cables; Long-eared Owl (*Asio otus*), use of pesticides and rodenticide, and collision with aerial cables and electrocution; Little Owl (*Athena noctua*), indiscriminate cutting of tree rows (especially mulberry trees (*Morus* sp.)), and impacts with overhead cables and wires or passing vehicles [6].

With the aim of protecting, rehabilitating, and analyzing these types of hazards, the wildlife rehabilitation center (WRC) plays a key role. Moreover, in Italy, Article 1 of Law 157/1992 states that: “Wildlife is the unavailable heritage of the State and is protected in the interest of the national and international community.” [7]. Regarding the European Union, Europe is home to more than 500 wild bird species. However, at least 32% of the EU’s bird species do not currently possess a good conservation status. The Birds Directive aims to protect all 500 wild bird species naturally occurring in the European Union. The main critical issues that motivated the legislation were the realization that wild bird species can only be protected through transboundary cooperation, as urban sprawl and transportation networks have fragmented and reduced their habitats, intensive agriculture, forestry, fishing, and pesticide use have diminished their food stocks, and hunting needed to be regulated so as not to harm populations. All of this convinced member states to unanimously adopt Directive 79/409/EEC in April 1979. This is the EU’s oldest piece of environmental legislation and one of its milestones. Amended in 2009, it became Directive 2009/147/EC [8].

Habitat loss and degradation are the most serious threats to the conservation of wild birds. The Directive therefore places great emphasis on the protection of habitats for endangered and migratory species. It establishes a network of Special Protection Areas (SPAs) including all of the most suitable territories for these species. Since 1994, all SPAs are included in the Natura 2000 ecological network, set up under the Habitats Directive 92/43/EEC [9].

There are almost 100 WRCs across Italy spread across all regions [10]. They are managed by animal rights associations or local authorities, without common data acquisition standards, treatment protocols, or release criteria. Consequently, a common national database is not available.

Data obtained from post-mortem carcass examinations or assessments of injured individuals admitted to rehabilitation centers are typically collected to analyze the cause of wildlife mortality, as it is economical and efficient in providing large samples over extensive geographical areas [11,12,13,14,15,16,17,18,19]. Therefore, these data are considered a useful means of understanding both the threats wild animal populations are facing and the mechanisms needed to monitor ecosystem health [20,21,22]. However, only a few studies on the causes of morbidity in wild raptors are available for the Italian territory [22]. Furthermore, long-term epidemiologic studies of wild raptor medical conditions spanning more than a decade are scarce worldwide [11,12,21,23,24,25,26,27,28]. Threats to raptors come mainly from humans and include habitat loss, climate change, poisoning, collisions, electrocutions, and deliberate capture or culling. In addition, other studies conducted on the causes of wild raptor hospitalizations report other causes such as orphans or young chicks mistakenly taken because they are presumed to have been abandoned by their parents or simply fallen from the nest, or infectious diseases (viral, bacterial, and parasitic). We performed an extensive literature search following PRISMA guidelines on the topic of the study, i.e., causes of admission of raptors to rehabilitation centers, without territorial limitations. We found a total of 46 publications; only one study covered Italy but included only one species, the long-eared owl (*Asio otus*). (Figure S1) These studies evaluated the causes of admission and assessed the outcome prognosis and origin of the injury.

The purpose of our 12-year retrospective study was to report the causes of admission of wild birds of prey received into the care of the WRC in Pescara ruled by the Carabinieri (formally “*Arma dei Carabinieri*”) Office for Biodiversity, Abruzzo, Italy, from 2005 to 2016. We added specific emphasis to factors associated with the reported diagnoses and the outcomes and the analysis of possible predictive indicators of survival and the possibility of release into the wild. This and other published retrospective studies provide critical information that can be used in the future to prevent and assess hazards affecting migratory and sedentary wild raptor populations, particularly in the Abruzzo region (central-southern Italy).

## 2. Materials and Methods

### 2.1. Study Area

Abruzzo is one of the 20 regions constituting the Italian State. It is located on the Adriatic side of central Italy. It is characterized by the prevalence of mountainous and hilly areas. It is divided into 4 provinces, namely, Chieti, Pescara, L’Aquila, and Teramo. In Abruzzo, there are 1,281,012 residents, 29.3% of which live in the province of Chieti, which covers 24.0% of the territory and where the population density is 144 inhabitants per km^2^. The province of Pescara, with 24.5% of residents but only 11.3% of the area, has 255 inhabitants per km^2^. Conversely, in L’Aquila, where 22.7% of residents occupy just under half of the regional territory, the population density is just 58 inhabitants per km^2^. Lastly, Teramo reported a population density of 121 inhabitants per km^2^. Abruzzo occupies fourteenth out of twenty in the ranking of population density per km^2^ in Italy, with an average of 118 inhabitants per km^2^ [29].

The territory of the region has been divided into 5 altitudinal classes: Plain 0–200 m above sea level (asl), hill > 200–400 m asl, high hills > 400–800 m asl, mountain > 800–1500 m asl, and high mountain > 1500 m asl, constituting 17.72%, 14.56%, 21.62%, 33.82%, and 12.28% of the regional territory, respectively [30].

Despite being a Mediterranean region, Abruzzo has some elements that divide the territory into two main climatic bands, characterized by large transition areas. The first of these elements is the Adriatic Sea, a basin with a depth less than that of the other seas, which therefore has a less balancing effect is and exposed to the cold and dry air masses coming from the Russian plateau. The second element consists of the eastern ridge of the central Apennines, such as the Laga, Gran Sasso, and Majella massifs and others, which must be considered under the dual profile of altitude and exposure [30].

Two bands can therefore be recognized: The first in the north-east, typical of the Adriatic Abruzzo, with a dominance of the Mediterranean climate, and the second to the south-west, more internal with mountain climatic characteristics [30].

This geographical characterization generates very strong climatic contrasts, which are also due to the poor balancing action of the Adriatic Sea, with very marked annual average excursions (between 17 °C and 18 °C), even in the sub-Apennine bands near the coast.

In Abruzzo, the following bioclimates are identifiable:

(1) Oceanic temperate climate. Typical of the Alps, the Apennines at high and medium altitudes and high-mountain Sicily. The climatic types vary from cryotemperate ultrahyperhumid-hyperhumid to hyperhumid-humid mesotemperate.
(2) Oceanic-semi-continental temperate climate. This is located in the central and eastern pre-Alps, in the hilly areas of the middle Adriatic, and in the internal valleys of the whole Apennines up to Basilicata with Tyrrhenian exposure. Local presence in Sardinia. The climatic types vary from supra-temperate/orotemperate to hyperhumid-ultrahyperhumid to humid sub-humid mesotemperate.
(3) Transitional oceanic temperate climate. This is located in all the valleys of the Tyrrhenian and Ionian anti-Apennines, with significant presence in the large islands. The climatic types vary from mesotemperate to humid/hyperhumid mesomediterranean.
(4) Oceanic-semicontinental transitional temperate climate. This is mainly located in the plains and the first hill buttresses of the middle and lower Adriatic and Ionian; significant presences in the inland areas of the Madonie and in some areas of Sardinia. Climatic types vary from sub-humid humid supratemperate to sub-humid humid mesomediterranean.
(5) Oceanic Mediterranean climate. This borders all of Italy from Liguria to Abruzzo to Pescara and the large islands. Climatic types vary from dry-subhumid inframediterranean to a subhumid thermo-Mediterranean [30].

**Figure 1:**
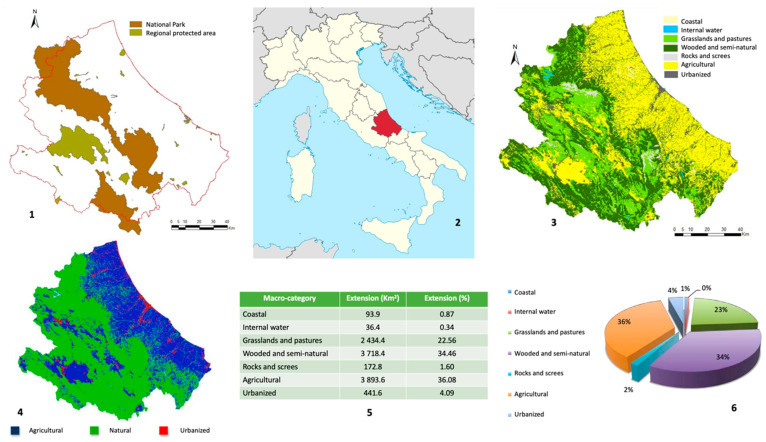
The main environmental characteristics of the Abruzzo region. Protected areas in the region (**1**); Abruzzo region in the Italian peninsula (**2**); environmental macro environmental categories (**3**); distribution of the type of environment within the region (**4**); percentage distribution of the 7 macro-categories of land use of the regional territory (**5**,**6**). (**2**) From https://it.wikipedia.org/wiki/Abruzzo#/media/File:Abruzzo_in_Italy.svg (accessed on 23 March 2020). (**1**,**3**–**6**) From “Istituto Superiore per la Ricerca e la Protezione Ambientale”—PIANO FAUNISTICO VENATORIO REGIONALE DELL’ABRUZZO 2019–2023. 2018.

Within its territories, there are many of Italy’s most precious national parks, including the “Abruzzo, Lazio and Molise National Park”, the “Majella National Park”, and the “Gran Sasso Monti della Laga National Park”. These data are shown in Figure 1.

### 2.2. Studied Population

All medical records were collected by veterinarians at the WRC of Pescara, ruled by the Carabinieri (formally “*Arma dei Carabinieri*”) Office for Biodiversity. These data were retrieved from records from the period of 2005 to 2016 and organized in annual Microsoft Excel files (Microsoft Corporation, Redmond, WA, USA). Only raptorial species were considered in the present study. The following information was extracted from the records: Species, date of discovery, date of admission and season (spring, summer, fall, and winter; according to the astronomical definition in the Northern Hemisphere), rescue location (province and locality), gender (if identifiable by external dimorphism, body size, or identification during postmortem examination), age (classified as fully feathered or not; this choice was made to avoid classification error during the 12 years studied), body weight at admission (in grams), and body condition score (good, medium, insufficient, and cachectic) assessed by the condition of the pectoral muscles [31].

Final or attempted diagnoses were made at the end of the rehabilitation process based on a physical examination performed by the attending veterinarian at admission, as well as the results of complementary examinations such as radiographs, hematology, cytology, toxicological analysis, and biomolecular investigations as needed. The following categories of diagnosis were recorded: Orphaned young nestling consisting of nestlings and fledglings supposedly abandoned by their parents or fallen from their nest (referred to simply as nestling in the following); starvation without other obvious primary causes; head trauma (including head injury, beak fracture or luxation, ocular and eyelid lesions); multiple trauma (fracture on more than one anatomical site); ischemic necrosis syndrome; thoracic limb trauma divided into the following categories: Fracture, luxation, and wound (gunshot or other traumatic wound); pelvic limb trauma (including fracture or luxation, as well as gunshot or other traumatic wound); traumatized plumage (sticky plants such as *Gallium aparine*, metal wire, or rodent glue); infectious and parasitic disease (blood or intestinal parasites; observation of external parasites in moderate quantities as reported in the medical record but not considered a cause of disease, except massive parasitosis or tick infestation); trunk trauma; toxic causes; illegal captivity; and undetermined cause. The diagnoses were further informed by the reported cause of admission, including nestling, starvation, trauma (at any anatomical site and for both hard and soft tissues), infectious or parasitic disease, toxic causes, indeterminate cause, illegal captivity, and dead-on-arrival (DOA).

Outcomes were classified into 4 categories: Release, euthanasia, death, and being kept in captivity. Regarding the last category, some of the raptors considered not releasable because survival in the wild was deemed highly unlikely were retained in captivity for teaching purposes and increasing awareness of environmental issues and wildlife protection.

### 2.3. Data Analysis

Data collected from medical records were coded numerically and organized in a table for further statistical analysis using Pivot graphics and tables in Excel 2016 (Microsoft Corporation, Redmond, WA, USA). The sex parameter was excluded from subsequent analysis because it was undetermined in more than half of the birds considered in the present study. First, to evaluate factors contributing to the cause of admission, a multinomial logistic regression model was performed with the cause of admission (excluding undetermined, since this category cannot be interpreted) as the dependent variable and species family (*Accipitridae*, *Falconidae*, *Tytonidae*, *Strigidae*), age (fully feathered or not), season, and year at the time of admission as independent variables.

To assess the evolution of the cause of admission over the years and within individual years, 2 multinomial logistic regression models were produced using the year and season of admission as dependent variables and the species, family, and age as independent variables.

Finally, to assess the presence of factors contributing to the rehabilitation outcome, a logistic regression model was performed considering the outcome (released vs. not released) as the dependent variable; other variables were the species family, the time between discovery and admission dates (GAP) (classified as zero, 1, between 2 and 3, between 4 and 10, or greater than 10 days) age, BCS, diagnosis (excluding ischemic necrosis syndrome as none of these birds were finally released, as well as undetermined causes and illegal captivity since describing birds without a true medical diagnosis would include uninterpretable results), season, and year of admission as independent variables. GAP and BCS are ordinal variables; they were first included categorically in the model and depending on whether the linearity of the coefficient was verified graphically, they were subsequently included as ordinal variables in the model.

Furthermore, the presence of an association between BCS and the cause of admission, as well as between BCS and GAP, was evaluated using a Kruskal–Wallis test. When the result of the Kruskal–Wallis test was significant, pairwise comparisons using Dunn’s procedure with Bonferroni correction for multiple comparisons were performed. The number of admissions and differences between years and between seasons were also evaluated using a chi-square test. All statistical tests, models, and odds ratios were considered significant at the alpha-type error level of 5%. The results of the different models are given with the p value (P) and the odds ratio (OR) followed by its 95% confidence interval.

All statistical tests and regression models were performed using IBM SPSS statistics software (IBM Corp. Released 2017. IBM SPSS Statistics 9 for Windows, Version 25.0. Armonk, NY, USA: IBM Corp.).

## 3. Results

### 3.1. Descriptive Analyses

During the 12-year study period, the WRC admitted a total of 3276 birds. Of them, 23.8% (780/3276) belonged to *Laridae*, *Columbidae*, *Sturnidae*, *Corvidae,* and other families; these birds were excluded from further analysis. The remaining 76.2% (2496/3276) of retrieved clinical reports concerned birds of prey species. Twenty-three different birds of prey species were admitted to the rehabilitation center. Of these, 48.8% (1219/2496) were nocturnal birds of prey, with 43.0% (1074/2496) belonging to the *Strigidae* family and 5.8% (145/2496) belonging to the *Tytonidae* family. Considering the diurnal species, 51.2% (1277/2496) of diurnal raptors were admitted, of which 20.8% (520/2496) belonged to the *Falconidae* family and 30.3% (757/2496) belonged to the *Accipitridae* family. All animals were admitted alive to the WRC, with only 0.6% (15/2496) DOA. The most common species were Little Owls (*Athena noctua*) (25.0%, 623/2496), followed by Common Buzzards (*Buteo buteo*) (20.3% 508/2496) and Common Kestrels (*Falco tinnunculus*) (17.4% 435/2496).

Raptors were classified either as plumage incompletely developed (23.3%, 581/2496) or as plumage completely developed (76.7%, 1915/2496). Concerning gender, 25.2% (628/2496) were recorded as males, 25.5% (637/2496) as females, and 49.3% (1323/2496) as undetermined.

For nocturnal raptors, trauma was the first cause of admission representing 45.8% (558/1219) of cases, followed by nestling (39.2%, 478/1219) and starvation (5.6%, 68/1219). Regarding diurnal raptors, trauma (73.1%, 934/1277), starvation (12.1%, 155/1277), and nestling (5.8%, 74/1277) were reported as the main causes of admission. Overall, the main causes of admission were trauma, nestling, and starvation (59.8%, 1492/2496; 22.1%, 552/2496; and 8.9%, 223/2496, respectively).

The overall WRC release rate during the study period was 39.4% (983/2496). The remaining birds (60.6%, 1513/2496) were distributed as follows: 39.4% (984/2496) died during treatment, 20.3% (506/2496) were euthanized, and 0.9% (23/2496) were deemed not releasable due to a permanent deficit for which they were kept in captivity for didactic purposes and awareness raising. The reader is referred to Table 1 for the remaining population characteristics included in this study.

### 3.2. Causes of Admission

Considering the low incidence of birds of prey cases admitted for infectious disease, intoxication, captivity, and dead-on-arrival, these categories were all grouped under a single label “other cause”, which was taken as the reference category in the multivariable multinomial regression model evaluating the risk factors associated with the different causes of admission during the study period. Table 2 reports the detailed descriptive statistics of these four causes of admission constituting the “other causes” new category.

The multivariable multinomial logistic regression model was significant and therefore considered interpretable (N = 2431, Chi^2^ = 1881.6, df = 108, *p* < 0.0001). Only results considered clinically relevant are commented upon and discussed. The reader is referred to Table S1 for the complete results of this model.

*Accipitridae* was significantly less common than *Falconidae* as nestlings compared to other causes of admission (infectious disease, intoxication, captivity, and DOA) (*p* = 0.043, OR = 0.427 [0.187–0.974]). As expected, birds of prey that presented as nestlings had significantly more commonly plumage not fully grown compared with birds that presented for other causes (*p* < 0.001, OR = 33.827 [18.533–61.745]). No raptor was admitted as a nestling during fall, and only one Tawny Owl was admitted presenting this admission cause at the end of winter (18 February). All other birds admitted as nestlings were presented during the first two seasons of the year, with 15.6% (86/552) of birds presenting in spring and 84.2% (465/552) in summer. Consequently, nestling was significantly more common in spring and summer than other causes of admission (*p* < 0.018, OR = 13.022 [1.550–109.438] and *p* < 0.007, OR = 17.855 [2.216–143.880], respectively).

*Strigidae* were significantly less common than *Falconidae* regarding starvation than for other causes (*p* = 0.008, OR = 0.451 [0.249–0.815]). Furthermore, significantly fewer raptors were admitted in fall than in winter for starvation than for other causes (*p* = 0.007, OR = 0.398 [0.205–0.775]).

Finally, *Strigidae* was significantly less frequently admitted than *Falconidae* for traumatism compared to other causes of admission (*p* = 0.014, OR = 0.565 [0.359–0.889]). Moreover, birds of prey admitted for traumatic causes were less frequently admitted in fall than in winter when compared to other causes of admission (*p* = 0.041, OR = 0.570 [0.332–0.977]). The raptors admitted for trauma more commonly presented with fully developed plumage (*p* = 0.009, OR = 0.475 [0.271–0.832]), which represented 94.6% (1411/1492) of all raptors admitted for traumatism.

BCS was found to be globally significantly different between causes of admission (Kruskal–Wallis test statistic = 195.6, ddl = 3, *p* < 0.001), with young birds presenting with significantly higher BCS and birds presenting with starvation having a significantly lower BCS (*p* < 0.001 for the two variables).

### 3.3. Annual and Seasonal Trends

**Figure 2:**
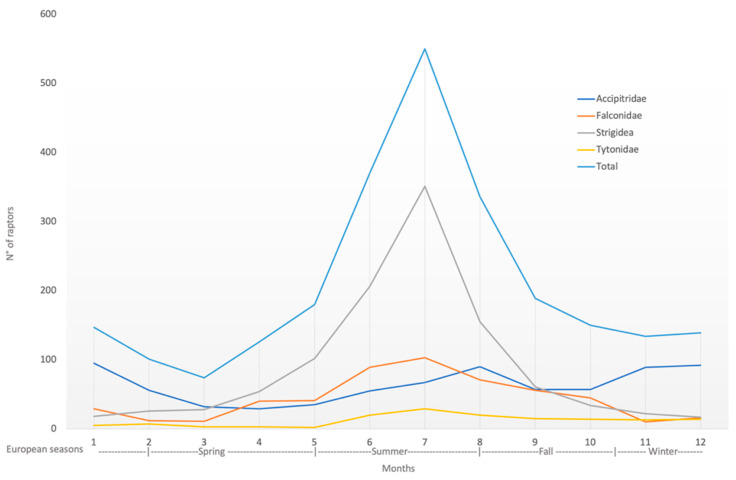
Seasonal overall trend of birds of prey admitted during the study period of 2005–2016, reported by families (*x*-axis shows the months of the year; *y*-axis shows the number of raptor specimens).

During the 12-year study period, 15.2% (380/2496) of birds of prey were admitted during spring, 50.3% (1256/2496) during summer, 19.0% (473/2496) during fall, and 15.5% (387/2496) during winter (Figure 2).

**Figure 3:**
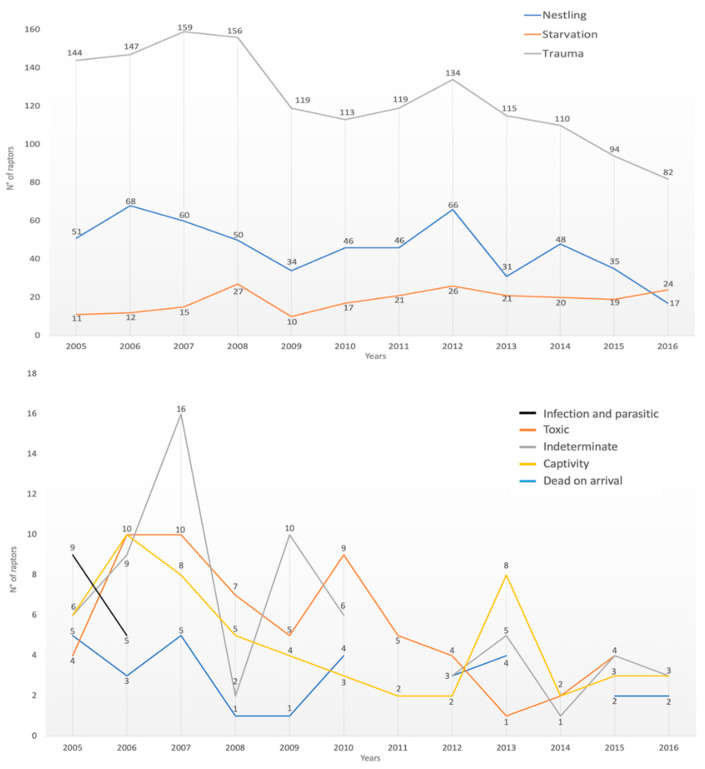
Number of cases for the different causes of admissions during the period of 1995–2007 as a function of the year of admission (*x*-axis shows the months of the year; *y*-axis shows the number of raptor specimens).

The causes of admission followed the overall annual trend of the cases, except for nestling, which, as seen above, had only one report in February and then reappeared from April to August; infectious/parasitic causes, which had zero cases in December and January; captivity birds of prey, with no cases reported for the months of February; and finally, DOA with no cases in February, April, September, October, and November (Figure 3).

### 3.4. Outcome and Release Rate

The logistic regression model that evaluated the outcome in relation to the year, family, GAP, season, year, age, BCS, and diagnosis was significant and therefore considered interpretable (N = 2181, Chi^2^ = 674.6, df = 36, *p* < 0.0001). Only results considered clinically relevant are discussed. The reader is referred to Table S2 for the complete results of this model. GAP was significantly associated with the outcome (*p* < 0.0001, OR = 1.23 [1.1–1.38]). Moving from one GAP category to the next, the birds were 1.2 times more likely to be released. Birds presented to the WRC in winter were significantly less frequently released than those presented in spring (*p* = 0.028, OR = 0.65 [0.44–0.96]. Furthermore, BCS was also significantly associated with the outcome (*p* < 0.0001, OR = 1.85 [1.61–2.13]). Moving from one BCS category to another, the birds had 1.8 times more chances of being released. The diagnosis with an overall better prognosis was nestling. In order of worsening prognosis, plumage trauma (OR = 0.39 [0.2–0.75]) and infectious/parasitic diseases (OR = 0.27 [0.12–0.62]), starvation (OR = 0.16 [0.1–0.27]), head trauma (OR = 0.16 [0.1–0.25]), and leg trauma (OR = 0.13 [0.07–0.24]) followed by wing trauma (luxation (OR = 0.1 [0.05–0.22]), wound (OR = 0.09 [0.04–0.2]), and fracture (OR = 0.08 [0.05–0.12])), and, finally, multiple trauma (OR = 0.05 [0.03–0.08]), intoxication (OR = 0.02 [0.01–0.06]), and trunk trauma (OR = 0.01 [0.001–0.08]) had the worst prognoses.

BCS was found to be globally significantly different between GAP categories (Kruskal–Wallis test statistic = 11.5, ddl = 4, *p* = 0.021) with a tendency to increase with increasing GAP but without a significant pairwise comparison in the post hoc procedure.

### 3.5. Diagnosis

Considering the more precise diagnosis, in *Strigidae* birds of prey, the most frequent diagnosis was nestling (40.2%, 432/1074), followed by head trauma (15.1%, 162/1074) and wing fracture (13.3%, 143/1074). For *Tytonidae*, the most frequent was wing fracture (29.7%, 43/145), followed by nestling (13.1%, 19/145) and multiple trauma (11.0%, 16/145). Regarding the diurnal species, the most common diagnosis for the family of *Accipitridae* was wing fracture (40.5%, 305/754), followed by starvation (14.1%, 106/754) and multiple trauma (10.2%, 77/754). For the *Falconidae* family, the most frequent diagnosis was wing fracture (30.2%, 158/523), followed by ischemic necrosis syndrome (23.9%, 125/523) and nestling (10.1%, 53/523). Overall, the most frequent diagnoses were wing fracture (26%, 649/2496) and nestling (21%, 524/2496), followed by head trauma (9.8%, 245/2496) and starvation (9.3%, 232/2496).

Glue-trapping was included in “traumatized plumage”; however, in detail, it represented 0.3% (8/2496) of cases admitted in the study period. Five Little Owls, One Barn Owl, and Two Common Kestrels were the species involved. A total of 0.6% (15/2496) of the gunshot wounds detected were admitted between October and February, except for one case admitted in July. All raptors with gunshot diagnoses belonged to diurnal raptor species, namely, 12 Common Buzzards, 1 Common Kestrel, and 2 Peregrine Falcons. Necrosis ischemic syndrome was found in *Falconidae* (72.3% of the total cases of necrosis ischemic syndrome, 125/173), and 21.4% (37/173) of the remaining cases belonged to the *Accipitridae* family. The main species involved was the Common Kestrel, with 99.2% (124/125) of *Falconidae* found to have necrosis ischemic syndrome belonging to this species.

## 4. Discussion

Descriptive epidemiological studies of wildlife are an important source of information about natural and non-natural hazards to wild animal populations. A long-term retrospective study on several raptor species in Italy is not available in the literature. The data presented in this retrospective study are based on 2496 birds of prey admitted to the Wildlife Rehabilitation Centre (WRC) of Pescara, ruled by Carabinieri (formally “*Arma dei Carabinieri*”). The analyzed data refer to a 12-year period, from 2005 to 2016. The raptors admitted belonged to 23 different species, which represent 57.5% of the raptor species present in Italian territories. Among the species treated in the WRC, several were of particular conservation importance and therefore classified as “*Vulnerable*” (VU) by the IUCN Italy: The Red-footed Falcon (*Falco vespertinus*), the Red Kite (*Milvus milvus*), the Western Marsh Harrier (*Circus aeruginosus*), Montagu’s Harrier (*Circus pygargus*), and the Short-toed Snake Eagle (*Circaetus gallicus*). Furthermore, another important species was the Griffon Vulture (*Gyps fulvus*), which is considered *“Critically Endangered*” (CR). This species in particular has been the subject of repopulation plans concerning the studied territories and is threatened by episodes of collective poisoning [32].

The most frequent causes of admission were trauma, nestling, and starvation. These results are consistent with those reported in previous retrospective studies in other countries [14,15,22,24,33]. In the present study, nestling of the *Accipitridae* family was less abundant than in *Falconidae*. The Common Buzzard and Eurasian Sparrowhawk represent the most frequent species of *Accipitridae*, while the Common Kestrel is the most represented species for *Falconidae* raptors. This observation could be explained by considering the type of nesting territory chosen by these species [4]. According to IUCN Italia, Common Buzzards are found to breed in a variety of habitats ranging from rocky cliffs and plateaus to woods and cultivated open fields [6]. In contrast, the Eurasian Sparrowhawk has a much narrower habitat, breeding in wooded territories or at their margins [6]. The Common Kestrel is often found in houses due to the tendency of this species to nest on top of chimneys or on the roof of country houses in rural environments, as well as in city bell towers and old masonry town buildings [6]. Therefore, Common Kestrels live in closer proximity to human beings, and nestlings are more likely to be found and presented to rehabilitation centers than Common Buzzards or Eurasian Sparrowhawks. However, 82% of the nestling in this study belonged to *Strigidae* species that typically leave the nest prematurely before they are fully fledged. The reason for this innate behavior might be to avoid parasitism and nest predators, or it could be due to human interference or the research for fresher perches [34]. These chicks, for the following months, still depend on the food procured by the parents and may appear helpless to well-meaning passers-by who collect them [21,22,35,36,37]. As reported by Mariacher et al. [22], the nestlings were usually healthy, uninjured chicks with good BCS, and rarely were they found in poor body condition, which is also consistent with the results of the present study; moreover, the release rate is usually the highest among all causes of admission as reported in a study conducted in a Hungarian WRC analyzing the Common Buzzard and Long-eared Owls [38].

As expected, according to the raptors’ breeding season for the latitudes, nestlings were admitted in spring and summer. No chicks of birds of prey were admitted during fall or winter except one Tawny Owl admitted in mid-February. This finding is considered normal for this species, since Tawny Owls are reported to breed in this territory from January to July, and the duration of the reproductive season, as well as its prolificacy, is influenced by two environmental factors: Prey abundance and duration of snow persistence on the ground [39].

Starvation represented 8.9% of total admissions, in line with other studies [11,12,14,22,40]. This cause of admission was reported to have a higher mortality rate in birds of prey during their first year of life [14,26,41]. This aspect is also confirmed in a study conducted in wildlife rescue centers in the Czech Republic where immature White-tailed Eagles (*Haliaeetus albicilla*) were less likely to be released than juvenile individuals [26].

Unfortunately, in our study, the age of the birds was not reported with enough precision to confirm this result in the present dataset. Fewer birds were admitted during the fall season than during winter, with starvation as the cause of admission. This could be explained by the presence of lower temperatures in winter, which require higher energy consumption to maintain body temperature [42]. This condition, associated with the possible presence of snow, adverse meteorologic conditions in the studied region [30,43], and reduction of prey availability, can lead a bird of prey to show cachexia even in the absence of evident detectable pathologies.

In the present study, birds of prey admitted with traumatic causes, wing trauma (fractures, luxation, and wounds), multi-trauma, and trunk trauma were associated with over 72% of negative outcomes. The authors agreed with the WRC veterinary staff who decided to analyze the exact anatomical site of the trauma. This decision was made because scarce information was obtained regarding the origin of various traumas, as most of the admission records lacked precise or reliable information on the finding or did not allow us to identify the exact primary origin of trauma. It was therefore chosen to keep information on the anatomical location of the trauma and not on its origin. This choice does not allow a full comparison of the origins of the trauma with other published studies; however, it was possible to confirm that the traumatic diagnosis is the main cause of admission for birds of prey in a WRC [13,14,15,26,27,28,40,44,45,46,47,48]. These results are, however, in contrast with finding trauma in only 5% of 92 free-living British Kestrels (*Falco tinnunculus*) in another study [49]. Overall, the most frequent diagnoses were wing fractures (26% of total admissions). Regarding the diurnal species, the most common diagnosis for *Accipitridae* and *Falconidae* raptors was represented by wing fracture (40.5% and 30.2% of the total diagnoses, respectively). *Strigidae* owls, excluding the nestling cause, reported head trauma (15.1%) and wing fracture (13.3%) as the most common trauma. *Tytonidae* reported wing fracture as the most frequent diagnosis (29.7%) and multiple trauma (11.0%). In particular, nocturnal raptors are prone to collision trauma and are highly vulnerable to the effects of road traffic. A valid and shared explanation for this vulnerability is that owls use roadside structures, such as trees, road signs, and fences, as support during their foraging habits, related to the abundance of small mammals on the roadsides; they may also suffer from temporary blindness when exposed to vehicle lights at night [6,22,24,50,51,52,53,54,55]. Connor T.P. et al. reported that urban areas were positively associated with persecution, building collisions, and unknown trauma admissions, whereas vehicle collisions were associated with more rural areas [28].

The low prevalence of infectious disease and toxicosis found in this study could be explained by the presence of multiple morbidity and mortality factors. These results were consistent with similar studies [15,21,24,25,28,56] but in contrast with those reported in Morishita et al. [13], who found that 30.3% (124/409) of the examined raptors had infectious or toxic effects. However, in this last study, only dead raptors presented for necropsy were analyzed, so neither nestling nor starvation of birds of prey were considered. Moreover, since the main aim of the WRC was to recover birds from their primary causes of admission, the role of underlying infectious or parasitic diseases could have been potentially underestimated because no complete microbiological and parasitological analyses were routinely performed on all the admitted raptors [22]. However, it is important to stress that the presence of sublethal infectious and toxic diseases (acute or chronic) could play a role in cases of traumatic causes of admission and act as a trigger or predisposing factor for birds to collision and other traumatic injuries, as reported by Molina-Lòpez et al. [24]. Therefore, birds of prey that presented with trauma may have had an underlying condition that could be not diagnosed due to the limited diagnostic tools available in the WRC, as well as because of the limited economic possibilities. In addition, only confirmed cases of infectious disease and toxicosis were listed in these categories. As reported in other studies, it is suggested that the prevalence of pesticide poisoning in raptors could be higher than actually reported [15,56,57]. Regarding this aspect, it is crucial to conduct a complete post-mortem examination as far as possible and, in the case of suspicion, conduct biomolecular investigations to search for specific pathogens (viral and bacterial pathologies) or contaminants and toxicity.

Summer was the season when more birds were admitted, which is consistent with reports in other wild rescue centers [12,15,16,33,44]. Autumn, winter, and spring were, in order, the seasons that followed according to the number of birds admitted. The month with the peak number of admissions in the summer season was July. *Strigidae* was responsible for this increment, particularly Little Owls. Birds admitted in winter were significantly less frequently released than those presented in spring. The authors’ hypothesis is that a bird seriously traumatized or in physical difficulty in winter is more likely to rapidly consume body energy reserves, considering the higher energy required to cope with the winter weather conditions [58]. Therefore, if found alive, raptors will more likely be admitted in winter with severe malnutrition in association with the primary pathology responsible for the problem.

The dynamics of cases during the study period showed a decrease in cases admitted to the center since 2009, except for 2012. The explanation for this fluctuation could be attributed to the devastating earthquake that hit the Abruzzo region and all of central Italy in April 2009. As a result of the earthquake, the tasks of WRC personnel changed, as did the accessibility of facilities.

GAP was significantly associated with the outcome. Indeed, WRC-admitted birds with a higher GAP were 1.2 times more likely to be released. This finding could be explained by only raptors with minor pathological conditions surviving for days without immediate treatment. Conversely, if a seriously ill raptor dies before being transferred to the WRC, it is registered as DOA or subjected to post-mortem examination at another national institution. BCS has also been significantly associated with the outcome, so it constitutes a prognostic index. As reported in other studies, a better BCS is more associated with a positive outcome and a higher post-release survival rate [24,25,59]. Our findings showed that, upon passing from one BCS category to the next, the birds had 1.8 times more chances of being released. These data provide a useful predictive indicator at the time of medical triage in admission to assess the chances of release into the wild. Greater opportunities and economic resources may be reserved for individuals with a better BCS (and thus greater chances of release). This indicator should be correlated and integrated with the percentages of release into the wild depending on the type of trauma (admission cause). From the evaluation of these two parameters, a scientific and valid prediction of that raptor’s chances of release into the wild can be established.

In the Abruzzo region, hunting is legally allowed from October to February. The 15 cases of gunshot trauma reported were admitted during those months, except for 1 case admitted in July. This result is confirmed by another study conducted in Rome, where birds were admitted almost exclusively during the hunting season. However, the authors of this study suggested that they were intentionally shot at during hunting activities, as birds of prey birds can be mistaken for game species, both in size and plumage, but also for flight characteristics [60].

No nocturnal raptors were found with this cause of admission, which could be explained by the nocturnal habits of owls reducing the chances of being shot. However, in a study focusing only on Long-eared Owls (*Asio otus*) conducted in Italy, gunshot injuries represented 4% of the cases in this nocturnal species [22]. Another study conducted in the LIPU (Lega Italiana Protezione Uccelli)/Bioparco wildlife rehabilitation center of Rome detected a higher incidence of gunshot birds admitted, compared to a similar study in Spain in 2011 [24,60].

In the Italian territory, all species of birds of prey are protected, and illegal hunting and detention are prohibited for the entire year, according to European Union Directive 2009/147/CE and national Italian laws (11 February 1992, N. 157) [7]. This result differs from what was found in Spain, where 9.6% of gunshot wounds (66/689) were detected outside of the hunting season [24]. One must consider the possibility of having areas where some risks are more expressed than others, so much so that they are the leading cause of reported admissions; this is the case of illegal killings in the Royal Society of the Conservation of Nature rehabilitation center in Jordan [61] and poisonings in Moholoholo Wildlife Rehabilitation Centre, Limpopo province, South Africa [62].

Ischemic necrosis syndrome is characterized by aseptic necrosis, with mummification and detachment of the distal extremities of the limbs (digit and wing). According to the etiopathogenetic hypothesis proposed by Delogu et al. [63], it is caused by thrombosis of the tarsal arterial vascular network and the bifurcation of the ulnar artery resulting from an infection with hemoparasites of the genus *Plasmodium* or *Haemoproteus* in immunosuppressed subjects [63,64,65]. This diagnosis was reported mainly in the Common Kestrel, similar to other published data [63,64,65] (71.7% of the total number of raptors with this diagnosis), and no birds of prey with this condition were ultimately released in this study, as for other cited reports [63,64,65].

A contrasting aspect with other studies is that no birds of prey presented lesions compatible with electrocution during the period studied [12,14,15,18,24,44,66,67]. This could be explained by the fact that unprotected medium-voltage (1–40 kV) powerlines are usually found in rural or sparsely populated areas [68]; this aspect reduces the possibility of finding electrocute birds. In addition, electrocuted birds of prey often die immediately or quickly become prey. Furthermore, the WRC of Pescara was not the competent authority dedicated to the collection and analysis of carcasses found in the area [12,14,68]. However, to completely investigate this threat to birds of prey, it would be necessary to evaluate not only the data coming from the WRCs but also to carry out a targeted and periodic census on lines particularly at risk [68].

Glue-trapping was included in “traumatized plumage”, with eight cases recorded in five Little Owls, one Barn Owl, and two Common Kestrels. In contrast to the report of Rodríguez et al. [12] in the WRC in Tenerife, the prognosis for this category of trauma was associated with higher mortality, with only two individuals released. Data confirmed the higher incidence in nocturnal raptors [12,44].

Regarding the release rate in the WRC, 39.4% of the admitted birds of prey were released. This percentage was comparable to other long-term retrospective studies available in the literature [24,32]. The causes of admission with better outcomes were nestling followed by indeterminate and infectious/parasitic (77.9%, 72.3%, and 60%, respectively). All others resulted in less than 40% positive outcomes (release). However, further studies and analysis of the data are needed to investigate the single outcome for the different specific diagnoses. Active post-release monitoring would be necessary to fully understand the criteria needed to achieve a successful release.

## 5. Conclusions

The results of this 12-year retrospective study highlighted the causes of admissions and the diagnoses reported at the WRC in Pescara ruled by the Carabinieri (formally “*Arma dei Carabinieri*”) Office for Biodiversity.

Critically ill birds must be assessed accurately and provided with immediate supportive care [69]. Specific protection and control plans to reduce anthropogenic causes of admissions, such as gunshots, use of pesticides and other toxic substances (with a direct effect on birds of prey, or indirect, i.e., on prey), and the use of rat glue, can and should be intensified in order to reduce these events to nil. Regarding the other causes related to urban and human activities, i.e., power grids, wind poles, impact with windows and glass, as well as with vehicles, deserve improved input data and specific studies, in order to better characterize individual areas (specific roads or specific territorial areas) to guide or at least sensitize public opinion and governmental authorities responsibly.

The rehabilitation and release of admitted birds to the wild can help buffer the negative effects of anthropogenic and non-human activities, especially for those species that have conservation concerns [28]. Further study needs to be conducted to improve and define predictive indicators of survival and the possibility of release into the wild as reported and emphasized in other studies [70,71,72]. As the data in our article show, the combination of BCS and trauma/admission cause can help provide a scientifically valid indication of the chances that a raptor has of being reintroduced to the wild, which we recall should be the ultimate goal of any WRC. These and other parameters, such as cause of admission, the severity of the injury, species (whether migratory or sedentary raptors), and age, may have a positive or negative effect on pre-release, as well as post-release, progression in the wild. For example, it is known that male sparrowhawks were less likely to be released than females [17], and that male raptors are more susceptible to spontaneous mortality than females [73]. Factors influencing survival rates before and after revival have been reviewed by Cope et al. [74] and should be used as a framework to guide rescue and treatment protocols, tailored to each species and based on global evidence of effectiveness. With these robust protocols in place, veterinarians and rescue organizations can continue to minimize animal suffering and maximize the effectiveness of rehabilitation programs in an increasingly anthropogenically expanding world with increasing climate change [74].

Another known parameter to take into account during the evaluation of the possibility for a raptor to be released is the length of stay in the WRC as reported for the Common Kestrels in the Czech Republic [72] and confirmed by Molony et al. [71].

One more consideration to make concerns the fate of non-recoverable animals. In fact, as it is reported [75], WRCs actively collaborate with Zoos and other organizations involved in the conservation of endangered species or for which reintroduction/repopulation projects are planned. In addition, to reduce costs and improve wildlife care, facilities such as zoos and teaching hospitals should be increasingly involved and integrated into the existing wildlife conservation system, subject to existing health and legal restrictions and limitations.

This first retrospective study, including 23 species of raptors in the Italian territory, provides critical information that can be used in the future to prevent and evaluate the dangers affecting migratory and resident wild raptor populations in the Abruzzo region of central Italy. This is of crucial importance, first because the Abruzzo region is home to resident and migratory species of national and international wildlife importance; and second because, although the Abruzzo region has a low population density and abundance of wild territories, causes of trauma have a significant impact on wild populations of both diurnal and nocturnal raptors.

## Figures and Tables

**Table 1:** Population characteristics of birds of prey admitted to the wildlife rehabilitation center of Pescara (Abruzzo, Italy) between 2005 and 2016, including causes of admission and release rate. IUCN classification and CITES status available at https://www.iucnredlist.org/ (accessed on 26 September 2020).

Common Name	Scientific Name	Cites Status—IUCN—Italy	Male/Female —Unknown	AGE (Plumage) Incomplete/Complete		Cause of Admission								OutcomeReleased (n/%)	Total Number
						Nestling	Starvation	Trauma	Infectious and Parasitic	Toxic	Undetermined	Captivity	Dead on Arrival		
**Family Accipitridae**															
**Montagu’ Harrier**	*Circus pygargus*	II/A—VU (LC Europe)	1/0—1	0	2	0	0	2	0	0	0	0	0	1/50%	2
**Western Marsh Harrier**	*Circus aeruginosus*	II/A—VU (LC Europe)	3/4—1	0	8	0	1	7	0	0	0	0	0	4/0%	8
**European Honey Buzzard**	*Pernis apivorus*	II/A—LC	0/8—13	0	21	0	3	16	0	0	1	1	0	6/29%	21
**Golden Eagle**	*Aquila chrysaetos*	II/A—NT (LC Europe)	1/4—0	0	5	0	2	3	0	0	0	0	0	2/40%	5
**Eurasian Sparrowhawk**	*Accipiter nisus*	II/A—LC	53/104—5	1	161	0	12	120	0	5	12	11	2	35/22%	162
**Northern Goshawk**	*Accipiter gentilis*	II/A—LC	10/17—6	3	30	2	2	23	0	2	3	0	1	15/45%	33
**Short-toed Snake Eagle**	*Circaetus gallicus*	II/A—VU (LC Europe)	1/2—1	0	4	0	2	2	0	0	0	0	0	3/75%	4
**Griffon Vulture**	*Gyps fulvus*	II/A—CR (LC Europe)	2/0—3	0	5	0	2	1	0	2	0	0	0	4/80%	5
**Red Kite**	*Milvus milvus*	II/A—VU (NT Europe)	3/0—2	0	5	0	1	4	0	0	0	0	0	2/40%	5
**Black Kite**	*Milvus migrants*	II/A—NT (LC Europe)	0/0—4	1	3	0	2	1	0	1	0	0	0	3/75%	4
**Common Buzzard**	*Buteo buteo*	II/A—LC	173/133—202	19	489	19	80	380	2	7	7	9	4	154/30%	508
**Family Falconidae**															
**Red-footed Falcon**	*Falco vespertinus*	II/A—VU (NT Europe)	1/3—0	0	4	0	1	3	0	0	0	0	0	1/25%	4
**Peregrine Falcon**	*Falco peregrinus*	I/A—LC	16/24—11	2	49	0	4	43	1	0	1	2	0	11/22%	51
**Common Kestrel**	*Falco tinnunculus*	II/A—LC	168/138—129	41	394	51	41	304	13	1	13	11	1	121/28%	435
**Lesser Kestrel**	*Falco naumanni*	II/A—LC	7/3—5	1	14	2	2	11	0	0	0	0	0	5/33%	15
**Eurasian Hobby**	*Falco subbuteo*	II/A—LC	4/5—4	0	13	0	0	12	0	0	0	1	0	2/15%	13
**Merlin**	*Falco columbarius*	II/A—NE (LC Europe)	2/0—0	0	2	0	0	2	0	0	0	0	0	0/0%	2
**Family Tytonidae**															
**Barn Owl**	*Tyto alba*	II/A—LC	37/41—67	24	121	20	14	91	2	5	7	5	1	57/39%	145
**Family Strigidae**															
**Tawny Owl**	*Strix aluco*	II/A—LC	18/38—103	79	80	75	4	73	1	1	2	2	1	97/61%	159
**Little Owl**	*Athene noctua*	II/A—LC	77/60—486	291	332	264	29	264	9	33	10	11	3	326/52%	623
**Long-eared Owl**	*Asio otus*	II/A—LC	37/39—57	33	100	27	9	81	2	7	3	3	1	48/36%	133
**Short-eared Owl**	*Asio flammeus*	II/A—NE (LC Europe)	1/0—0	0	1	0	0	1	0	0	0	0	0	0/0%	1
**Eurasian Scops Owl**	*Otus scops*	II/A—LC	13/14—131	86	72	92	12	48	0	0	6	0	0	86/54%	158

**Table 2:** Descriptive statistics for each cause of admission included under the label “other causes of admission” (infectious disease, intoxication, captivity, and dead-on-arrival (DOA)).

Admission Cause	Infectious			Toxic			Captivity			DOA		
	Total Admitted	N° with This Cause	Percentage (%)	Total Admitted	N° with This Cause	Percentage (%)	Total Admitted	N° with This Cause	Percentage (%)	Total Admitted	N° with This Cause	Percentage (%)
**Family**												
Strigidae	1053	12	1.1	1053	41	3.9	1053	16	1.5	1053	5	0.5
Tytonidae	138	2	1.4	138	5	3.6	138	5	3.6	138	1	0.7
Accipitridae	731	2	0.3	731	15	2.1	731	21	2.9	731	7	1
Falconidae	509	14	2.8	509	3	0.6	509	14	2.8	509	1	0.2
**Year**												
2005	230	5	2.2	230	4	1.7	230	6	2.6	230	9	3.9
2006	255	3	1.2	255	10	3.9	255	10	3.9	255	5	2
2007	257	5	1.9	257	10	3.9	257	8	3.1	257	0	0
2008	246	1	0.4	246	7	2.8	246	5	2	246	0	0
2009	173	1	0.6	173	5	2.9	173	4	2.3	173	0	0
2010	192	4	2.1	192	9	4.7	192	3	1.6	192	0	0
2011	193	0	0	193	5	2.6	193	2	1	193	0	0
2012	235	3	1.3	235	4	1.7	235	2	0.9	235	0	0
2013	180	4	2.2	180	1	0.6	180	8	4.4	180	0	0
2014	182	0	0	182	2	1.1	182	2	1.1	182	0	0
2015	157	2	1.3	157	4	2.5	157	3	1.9	157	0	0
2016	131	2	1.5	131	3	2.3	131	3	2.3	131	0	0
**Age (plumage)**												
Incomplete	592	4	0.7	592	8	1.4	592	6	1	592	3	0.5
Complete	1839	26	1.4	1839	56	3	1839	50	2.7	1839	11	0.6
**Season**												
Spring	365	3	0.8	365	9	2.5	365	10	2.7	365	3	0.8
Summer	1235	15	1.2	1235	28	2.3	1235	19	1.5	1235	7	0.6
Fall	456	11	2.4	456	17	3.7	456	20	4.4	456	0	0
Winter	375	1	0.3	375	10	2.7	375	7	1.9	375	4	1.1

## Data Availability

The data used for the study were made available by the State Forestry Corps.

## Supplementary Materials

The following supporting information can be downloaded at: https://www.mdpi.com/article/10.3390/ani12151916/s1, Figure S1: PRISMA flowchart literature review on the topic: Causes of admission to a raptor/birds of prey rehabilitation/rescue center, including the list of 46 publication finally selected during the literature search process. (Updated June 2022); Table S1: Complete data obtained from the multivariable multinomial logistic regression model. Which was significant and therefore considered interpretable (N = 2431, Chi^2^ = 1881.6, df = 108, *p* < 0.0001); Table S2: The complete data of the logistic regression model that evaluated the outcome in relation to the year, family, GAP, season, year, age, BCS, and diagnosis was significant and therefore considered interpretable (N = 2181, Chi^2^ = 674.6, df = 36, *p* < 0.0001).
