# Supplementary material for: Causes of Raptor Admission to the Wildlife Rehabilitation Centre in Abruzzo (Central Italy) from 2005–2016
Source: Animals (Basel). 2022 Jul 27;12(15):1916. doi: 10.3390/ani12151916 (PMC9367453; doi:10.3390/ani12151916)
Supplement: Supplementary file 1 [file animals-12-01916-s001.zip › animals-1746303-supplementary.pdf]

Figure S1: PRISMA flowchart literature review on the topic: Causes of admission to a raptor/birds of prey rehabilitation/rescue center, including the list of 46 publication finally selected during the literature search process. (Updated June 2022)

Search strategy

Searches in electronic databases and PDFs (proceedings, textbooks, etc.) were performed based on a combination of keywords for the target condition.

Published literature- The following electronic databases were consulted: MEDLINE (Pubmed), Google scholar, ScienceDirect.

Grey literature- To explore the grey literature (i.e., non-published material) conference proceedings, grey databases and textbooks were searched.

- The conference proceedings of the following conferences were searched: ExoticsCon conference, International Conference on Avian, Herpetological and Exotic Mammal Medicine (ICARE) (from 2013 to 2019).
- The following grey databases were searched: Open Grey, and VIN (Veterinary Information Network).

Search procedures

Electronic searches (databases): The searches on databases were planned to maximize sensitivity. The Boolean operator 'AND' was used between the key concepts, and English language only was selected.

(Admission causes) AND ((raptor) OR (birds of prey))

Refined search adding (long-term study) and (more than on species of bird).

Inclusion criteria

*Study designs-*Retrospective study, aiming to analyze the admission causes of wild population of raptors/birds of prey.

Participants- Bird of preys – raptors – one species - more species – nocturnal and diurnal raptors. All species, subspecies, age, and sex were eligible for inclusion.

First target study- The target study was found long-term study conducted in Italy analyzing the admission causes in wild rehabilitation center for raptors/birds of prey.

Second Target study: selected the literature conserving wildlife rehabilitation center analyzing raptors and the causes of admission.

Results

The search of databases provided a total of 36828 citations after the improved terminology used to search.

The key words were adjusted to obtain results more specific. However, article related to the releasing and post releasing monitoring were considered.

- Pubmed: 23 results obtained – improved research 5 - 5 eligible.
- Science Direct: 93 – improved research 3 – only 3 articles and 1 book – 4 eligible.
- Google scholar: 36700 – 38 eligible.

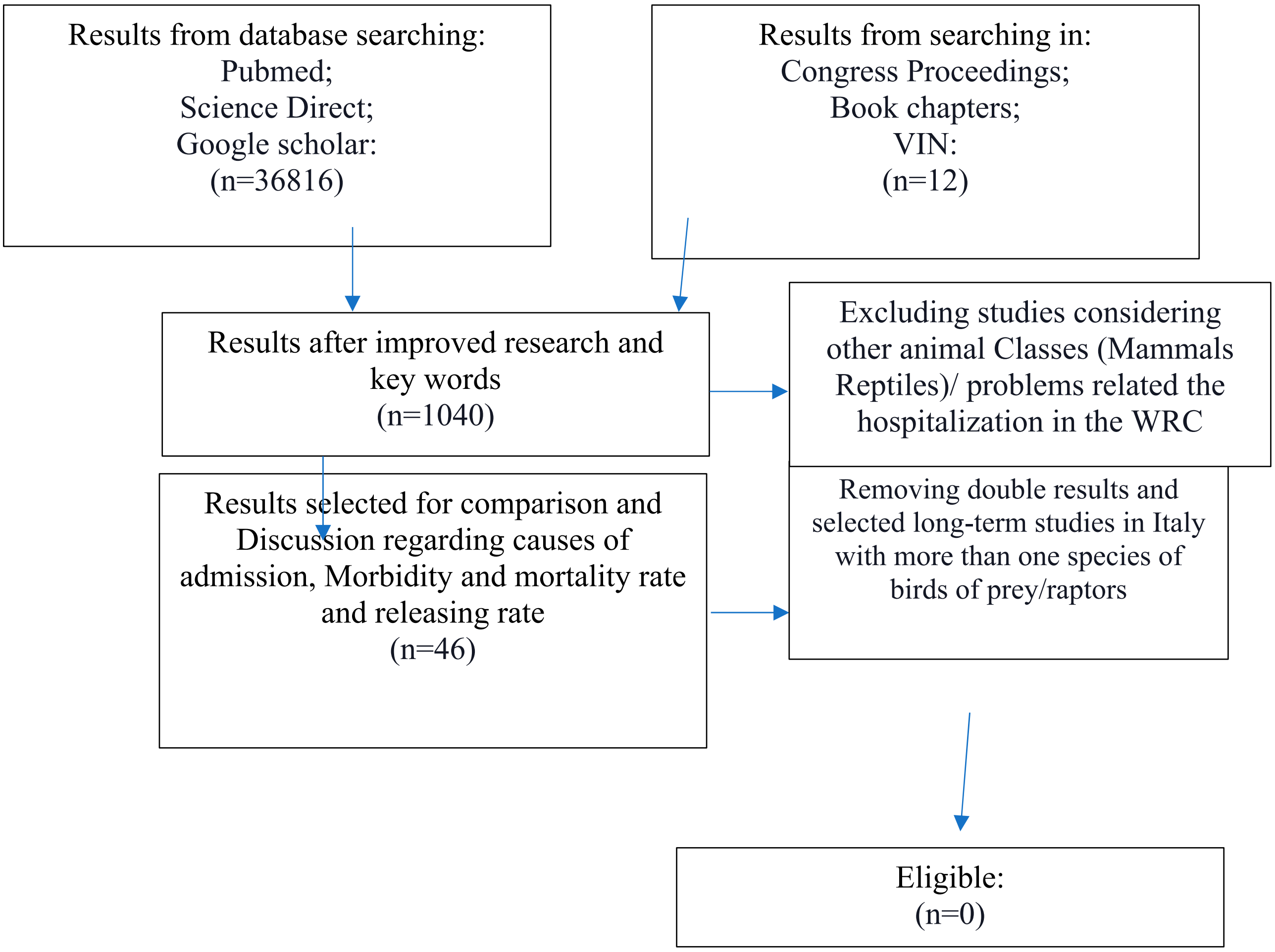

Please note that, the result of this literature research was updating several times until June 2022

1. Kovács, A.; Mammen, U.C.C.; Wernham, C.V. European monitoring for raptors and owls: State of the art and future needs. *Ambio* **2008**, *37*, 408–412; DOI:10.1579/0044-7447(2008)37[408:emfrao]2.0.co;2.
2. Burfield, I.J. The conservation status and trends of raptors and owls in Europe. *Ambio* **2008**, *37*, 401–407; DOI:10.1579/0044-7447(2008)37[401:tcsato]2.0.co;2.
3. Newton, I.; Wyllie, I.; Dale, L. Trends in the numbers and mortality patterns of sparrowhawks (*Accipiter nisus*) and kestrels (*Falco tinnunculus*) in Britain, as revealed by carcass analyses. *J. Zool.* **1999**, *248*, 139–147; DOI:10.1111/j.1469-7998.1999.tb01190.x.
4. Rodríguez, B.; Rodríguez, A.; Siverio, F.; Siverio, M. Causes of raptor admissions to a wildlife rehabilitation center in Tenerife (Canary Islands). *J. Raptor Res.* **2010**, *44*, 30–39; DOI:10.3356/jrr-09-40.1.
5. Work, T.M.; Hale, J. Causes of owl mortality in Hawaii, 1992 to 1994. *J. Wildl. Dis.* **1996**, *32*, 266–273; DOI:10.7589/0090-3558-32.2.266.
6. Morishita, T.Y.; Fullerton, A.T.; Lowenstine, L.J.; Gardner, I.A.; Brooks, D.L. Morbidity and mortality in free-living raptorial birds of Northern California: A retrospective study, 1983-1994. *J. Avian Med. Surg.* **1998**, *12*, 78–81.
7. Wendell, M.D.; Sleeman, J.M.; Kratz, G. Retrospective study of morbidity and mortality of raptors admitted to Colorado state university veterinary teaching hospital during 1995 to 1998. *J. Wildl. Dis.* **2002**, *38*, 101–106; DOI:10.7589/0090-3558-38.1.101.
8. Martínez, J.A.; Martínez, J.E.; Mañosa, S.; Zuberogoitia, I.; Calvo, J.F. How to manage human-induced mortality in the eagle owl *Bubo bubo*. *Bird Conserv. Int.* **2006**, *16*, 265–278; DOI:10.1017/s0959270906000402.
9. Kelly, A.; Bland, M. Admissions, diagnoses, and outcomes for Eurasian sparrowhawks (*Accipiter nisus*) brought to a wildlife rehabilitation center in England. *J. Raptor Res.* **2006**, *40*, 231–235; DOI:10.3356/0892-1016(2006)40[231:adaofe]2.0.co;2.
10. Margalida, A.; Heredia, R.; Razin, M.; Hernández, M. Sources of variation in mortality of the bearded vulture *Gypaetus barbatus* in Europe. *Bird Conserv. Int.* **2008**, *18*, 1–10; DOI:10.1017/s0959270908000026.
11. Molina, R.A.; Darwich, L. Causes of admission of little owl (*Athene noctua*) at a wildlife rehabilitation centre in Catalonia (Spain) from 1995 to 2010. *Anim. Biodivers. Conserv.* **2011**, *34*, 401–405.
12. Mariacher, A.; Gherardi, R.; Mastrorilli, M.; Melini, D. Causes of admission and outcomes of long-eared owl (*Asio otus*) in wildlife rescue centres in Italy from 2010 to 2014. *Avian Biol. Res.* **2016**, *9*, 282–286; DOI:10.3184/175815516x14739467542487.
13. Harris, M.C.; Sleeman, J.M. Morbidity and mortality of bald eagles (*Haliaeetus leucocephalus*) and peregrine falcons (*Falco peregrinus*) admitted to the wildlife center of Virginia, 1993–2003. *J. Zoo Wildl. Med.* **2007**, *38*, 62–66; DOI:10.1638/05-099.1.
14. Molina-López, R.A.; Casal, J.; Darwich, L. Causes of morbidity in wild raptor populations admitted at a wildlife rehabilitation centre in Spain from 1995-2007: A long term retrospective study. *PLoS One* **2011**, *6*, e24603; DOI:10.1371/journal.pone.0024603.
15. Molina-López, R.A.; Mañosa, S.; Torres-Riera, A.; Pomarol, M.; Darwich, L. Morbidity, outcomes and cost-benefit analysis of wildlife rehabilitation in Catalonia (Spain). *PLoS One.* **2017**, *12*, e0181331; DOI:10.1371/journal.pone.0181331.
16. Rozsypalová, L.; Rymešová, D.; Stýblo, P.; Literák, I. Causes of admission and outcomes of white-tailed eagles Haliaeetus albicilla in wildlife rescue centres in the Czech Republic during 2010–2020. *Avian Biol. Res*. **2022**, DOI:10.1177/17581559221107083.
17. Mazaris, A. D.; Mamakis, Y.; Kalpakis, S.; Poulopoulos, Y.; Matsinos, Y. G. Evaluating potential threats to birds in Greece: An analysis of a 10-year data set from a rehabilitation centre. *Oryx*, **2008** , 42, 408– 414. DOI:10.1017/S003060530700066X
18. Panter, C.T.; Allen, S.; Backhouse, N.; Mullineaux, E.; Rose, C.-A.; Amar, A. Causes, temporal trends, and the effects of urbanization on admissions of wild raptors to rehabilitation centers in England and Wales. *Ecol. Evol.* **2022**, 12:e8856. DOI:10.1002/ece3.8856.
19. Montesdeoca, N.; Calabuig, P.; Corbera, J.A.; Orós, J. A long-term retrospective study on rehabilitation of seabirds in Gran Canaria Island, Spain (2003-2013). *PLoS One* **2017**, *12*, e0177366; DOI:10.1371/journal.pone.0177366.
20. Petty, S.J.; Thirgood, S.J. A radio tracking study of post-fledging mortality and movements of Tawny Owls in Argyll. *Ringing Migr.* **1989**, *10*, 75–82; DOI:10.1080/03078698.1989.9673943.
21. Griffiths, R.; Murn, C.; Clubb, R. Survivorship of rehabilitated juvenile Tawny Owls (*Strix aluco*) released without support food, a radio tracking study. *Avian Biol. Res.* **2010**, *3*, 1–6; DOI:10.3184/175815510x12628917082461.
22. Sós-Koroknai, V.; Gál, J.; Marosán, M.; Tóth, T. Causes of morbidity of common buzzards (*Buteo buteo*) and long-eared owls (*Asio otus*) at a Hungarian wildlife rescue centre between 2014 and 2016. *Biharean Biol*. **2020** Jun 1;14(1):25-9.
23. Pavez, E.F.; Estades, C.F. Causes of admission to a rehabilitation center for andean condors (*Vultur gryphus*) in Chile. *J. Raptor Res.* **2016**, *50*, 23–32; DOI:10.3356/rapt-50-01-23-32.1.
24. Montesdeoca, N.; Calabuig, P.; Corbera, J.A.; Orós, J. Causes of admission for raptors to the Tafira wildlife rehabilitation center, Gran Canaria Island, Spain: 2003–13. *J. Wildl. Dis.* **2016**, *52*, 647; DOI:10.7589/2015-09-255.
25. Coon, N.C.; Locke, L.N.; Cromartie, E.; Reichel, W.L. Causes of bald eagle mortality, 1960–1965. *J. Wildl. Dis.* **1970**, *6*, 72–76; DOI:10.7589/0090-3558-6.1.72.

26. Fix, A.S.; Barrows, S.Z. Raptors rehabilitated in Iowa during 1986 and 1987: A retrospective study. *J. Wildl. Dis.* **1990**, *26*, 18–21; DOI:10.7589/0090-3558-26.1.18.
27. Deem, S.L.; Terrell, S.P.; Forrester, D.J. A retrospective study of morbidity and mortality of raptors in Florida: 1988-1994. *J. Zoo Wildl. Med.* **1998**, *29*, 160–164.
28. Stewart, P.A. Movements, population fluctuations, and mortality among great horned owls. *Wilson Bull.* **1969**, *81*, 155–162.
29. Keymer, I.F.; Fletcher, M.R.; Stanley, P.I. Causes of mortality in British kestrels (*Falco tinnunculus*). In *Recent Advances in the Study of Raptor Diseases*; Chiron, **1980**; pp. 143–151.
30. Erickson, W.P.; Johnson, G.D.; Young, D.P.J. A summary and comparison of bird mortality from anthropogenic causes with an emphasis on collisions. Ralph C John Rich Terrell Ed 2005 Bird Conserv Implement Integr Am Proc Third Int Partn Flight Conf 2002 March 20-24 Asilomar Calif Vol 2 Gen Tech Rep PSW-GTR-191 Albany CA US Dept Agric For Serv Pac Southwest Res Stn P 1029-1042. 2005;191. Available online: https://www.fs.usda.gov/treesearch/pubs/32103 (accessed on 30 November 2020).
31. Erritzøe, J. Causes of mortality in the long-eared *owl Asio otus*. *Dan. Ornitol. Foren. Tidskr.* **1999**, *93*, 162–164.
32. Erritzoe, J.; Mazgajski, T.D.; Rejt, Ł. Bird casualties on European roads — A review. *Acta Ornithol.* **2003**, *38*, 77–93; DOI:10.3161/068.038.0204.
33. Massemin, S.; Zorn, T. Highway mortality of barn owls in north-eastern France. *J. Raptor Res.* **1998**, *32*, 229–232.
34. Gottdenker, N.L.; Walsh, T.; Jiménez-Uzcátegui, G.; Betancourt, F.; Cruz, M.; Soos, C.; Miller, R.E.; Parker, P.G. Causes of mortality of wild birds submitted to the charles darwin research station, Santa Cruz, Galápagos, Ecuador from 2002–2004. *J. Wildl. Dis.* **2008**, *44*, 1024–1031; DOI:10.7589/0090-3558-44.4.1024.
35. Molina-López, R.A.; Casal, J.; Darwich, L. Prognostic indicators associated with early mortality of wild raptors admitted to a wildlife rehabilitation centre in Spain. *Vet. Q.* **2015**, *35*, 9–15; DOI:10.1080/01652176.2014.985856.
36. Cianchetti-Benedetti M.; Manzia F.; Fraticelli F.; J. G. Cecere. Shooting is still a main threat for raptors inhabiting urban and suburban areas of Rome, Italy, *Ital. J. Zool*. **2016**, 83:3, 434-442, DOI: 10.1080/11250003.2016.1189611.
37. Mohammad Y.; Al Zoubi,; Nashat A.; Hamidan, Mohammad A.; Abu Baker,; Zuhair Amr. Causes of Raptor Admissions to Rehabilitation in Jordan. *J Raptor Res* **2020**; 54 (3): 273–278. DOI: 10.3356/0892-1016-54.3.273
38. Mashele N.M.; Thompson L.J.; Downs C.T. Trends in the admission of raptors to the Moholoholo Wildlife Rehabilitation Centre, Limpopo province, South Africa, *Afr Zool*. **2022,** 57:1, 56-63, DOI: 10.1080/15627020.2021.2016073
39. Desmarchelier, M.; Santamaria-Bouvier, A.; Fitzgérald, G.; Lair, S. Mortality and morbidity associated with gunshot in raptorial birds from the province of Quebec: 1986 to 2007. *Can. Vet. J.* **2010**, *51*, 70–74.
40. Hager, S.B. Human-related threats to urban raptors. *J. Raptor Res.* **2009**, *43*, 210–226; DOI:10.3356/jrr-08-63.1.
41. Maphalala, MI.; Monadjem, A.; Bildstein, KL.; Hoffman, B.; Downs, C. Causes of admission to a raptor rehabilitation centre and factors that can be used to predict the likelihood of release. *Afr J Ecol*. **2021**, 59: 510– 517. DOI:10.1111/aje.12851.
42. Molony, SE.; Baker, PJ.; Garland, L.; Cuthill, IC.; Harris, S. Factors that can be used to predict release rates for wildlife casualties. *Anim. Welfare* **2007,** 16, 361–367.
43. Lukesova, G.; Voslarova, E.; Vecerek, V.; Vucinic M. Causes of admission, length of stay and outcomes for common kestrels in rehabilitation centres in the Czech Republic. *Sci Rep* **2021**,11, 17269. DOI:10.1038/s41598-021-96688-8.
44. Kelly A.; Bland M. Admissions, diagnoses, and outcomes for Eurasian Sparrowhawks (*Accipiter nisus*) brought to a wildlife rehabilitation center in England. *J Raptor Res*. **2006**;40(3):231–5. DOI: 10.3356/0892-1016(2006)40[231:Adaofe]2.0.Co;2]
45. Molina-López R.A.; Casal J.;, Darwich L. Final disposition and quality auditing of the rehabilitation process in wild raptors admitted to a wildlife rehabilitation centre in Catalonia, Spain, during a twelve year period (1995–2007). *PLoS ONE*. **2013**;8(4):e60242. DOI: 10.1371/journal.pone.0060242.
46. Cope H.R.; McArthur C.; Dickman C.R.; Newsome T.M.; Gray R.: Herbert C.A. A systematic review of factors affecting wildlife survival during rehabilitation and release. *PLoS One*. **2022** Mar 17;17(3):e0265514. DOI: 10.1371/journal.pone.0265514.

Table S1: Multivariable multinomial logistic regression model evaluating risk factors for the different causes of admission in free-ranging birds of prey admitted at the Wildlife Rehabilitation Center of Pescara (Abruzzo, Italy) from 2005 to 2016 (the causes of admission infectious disease, intoxication, captivity by citizens and dead-on-arrival were reunited under the label "other causes of admission" considering the few number of admissions for each separated cause during the study period). Highlighted lines correspond to significant results.

Table S2: Multivariable logistic regression model evaluating factors influencing the outcome of birds of prey admitted to the Wildlife Rehabilitation Center of Pescara (Abruzzo, Italy) from 2005 to 2016. Highlighted lines correspond to significant results.

Table S1.

Admission cause		Total N° of admitted	N° of admitted	% N of admitted	Odds ratio	Confidence interval 95 %		P-Value
						Lower	Upper	
Nestling	**Family**							
	Strigidae	1053	458	43.5	0.909	0.487	1.695	0.764
	Tytonidae	138	20	14.5	0.535	0.184	1.554	0.250
	Accipitridae	731	21	2.9	0.427	0.187	0.974	0.043
	Falconidae	509	53	10.4	Referent			
	**Year**							
	2005	230	51	22.2	2.373	0.764	7.372	0.135
	2006	255	68	26.7	2.236	0.742	6.739	0.153
	2007	257	60	23.3	2.924	0.943	9.067	0.063
	2008	246	50	20.3	3.837	1.169	12.592	0.027
	2009	173	34	19.7	2.869	0.812	10.134	0.102
	2010	192	46	24.0	6.148	1.829	20.662	0.003
	2011	193	46	23.8	6.926	1.845	25.999	0.004
	2012	235	66	28.1	7.376	2.114	25.741	0.002
	2013	180	31	17.2	1.737	0.517	5.832	0.372
	2014	182	48	26.4	17.056	3.902	74.544	<0.001
	2015	157	35	22.3	5.440	1.469	20.146	0.011
	2016	131	17	13.0	Referent			
	**Age (plumage)**							
	Incomplete	592	492	83.1	33.827	18.533	61.745	<0.001
	Complete	1839	60	3.3	Referent			
	**Season**							
	Spring	365	86	23.6	13.022	1.550	109.438	0.018
	Summer	1235	465	37.7	17.855	2.216	143.880	0.007
	Fall	456	0	0.0	NC	NC	NC	NC
	Winter	375	1	0.3	Referent			
Starvation	**Family**							
	Strigidae	1053	54	5.1	0.451	0.249	0.815	0.008
	Tytonidae	138	14	10.1	0.898	0.363	2.220	0.815
	Accipitridae	731	105	14.4	1.543	0.856	2.781	0.149
	Falconidae	509	50	9.8	Referent			
	**Year**							

	2005	230	11	4.8	0.132	0.044	0.393	<0.001
	2006	255	12	4.7	0.132	0.045	0.385	<0.001
	2007	257	15	5.8	0.173	0.060	0.498	0.001
	2008	246	27	11.0	0.661	0.229	1.907	0.444
	2009	173	10	5.8	0.333	0.099	1.113	0.074
	2010	192	17	8.9	0.289	0.098	0.853	0.025
	2011	193	21	10.9	0.921	0.278	3.044	0.892
	2012	235	26	11.1	0.844	0.274	2.600	0.768
	2013	180	21	11.7	0.485	0.164	1.429	0.189
	2014	182	20	11.0	1.598	0.409	6.246	0.500
	2015	157	19	12.1	0.661	0.208	2.097	0.482
	2016	131	24	18.3	Referent			
	**Age (plumage)**							
	Incomplete	592	16	2.7	0.673	0.315	1.439	0.307
	Complete	1839	207	11.3	Referent			
	**Season**							
	Spring	365	25	6.8	0.543	0.248	1.188	0.127
	Summer	1235	103	8.3	0.902	0.478	1.701	0.750
	Fall	456	42	9.2	0.398	0.205	0.775	0.007
	Winter	375	53	14.1	Referent			
Trauma	**Family**							
	Strigidae	1053	467	44.3	0.565	0.359	0.889	0.014
	Tytonidae	138	91	65.9	0.710	0.352	1.429	0.337
	Accipitridae	731	560	76.6	1.046	0.642	1.703	0.857
	Falconidae	509	374	73.5	Referent			
	**Year**							
	2005	230	144	62.6	0.494	0.208	1.175	0.111
	2006	255	147	57.6	0.457	0.195	1.071	0.072
	2007	257	159	61.9	0.555	0.233	1.324	0.184
	2008	246	156	63.4	1.057	0.414	2.700	0.908
	2009	173	119	68.8	1.074	0.400	2.888	0.887
	2010	192	113	58.9	0.553	0.221	1.388	0.207
	2011	193	119	61.7	1.486	0.509	4.338	0.469
	2012	235	134	57.0	1.247	0.455	3.421	0.668
	2013	180	115	63.9	0.759	0.295	1.951	0.567
	2014	182	110	60.4	2.403	0.687	8.404	0.170
	2015	157	94	59.9	0.864	0.312	2.396	0.779
	2016	131	82	62.6	Referent			
	**Age (plumage)**							
	Incomplete	592	81	13.7	0.475	0.271	0.832	0.009
	Complete	1839	1411	76.7	Referent			
	**Season**							

	Spring	365	229	62.7	0.800	0.427	1.497	0.484
	Summer	1235	598	48.4	0.811	0.474	1.387	0.444
	Fall	456	366	80.3	0.570	0.332	0.977	0.041
	Winter	375	299	79.7	Referent			
Other causes	**Family**				Referent			
	Strigidae	1053	74	7.0				
	Tytonidae	138	13	9.4				
	Accipitridae	731	45	6.2				
	Falconidae	509	32	6.3				
	**Year**							
	2005	230	24	10.4				
	2006	255	28	11.0				
	2007	257	23	8.9				
	2008	246	13	5.3				
	2009	173	10	5.8				
	2010	192	16	8.3				
	2011	193	7	3.6				
	2012	235	9	3.8				
	2013	180	13	7.2				
	2014	182	4	2.2				
	2015	157	9	5.7				
	2016	131	8	6.1				
	**Age (plumage)**							
	Incomplete	592	21	3.5				
	Complete	1839	143	7.8				
	**Season**							
	Spring	365	25	6.8				
	Summer	1235	69	5.6				
	Fall	456	48	10.5				
	Winter	375	22	5.9				

Table S2.

	Admitted	Released	Released rate (%)	Odds ratio	Confidence interval 95%		P-value
					Lower	Upper	
**FAMILY**							
Strigidae	1029	541	52.6	Referent			
Tytonidae	125	51	40.8	1.491	0.946	2.348	0.085
Accipitridae	657	193	29.4	0.995	0.752	1.317	0.973
Falconidae	370	127	34.3	0.896	0.654	1.228	0.495
**GAP**				1.232	1.099	1.381	< 0.001
0	972	380	39.1	Linear correlation			
1	795	329	41.4				
2 to 3	306	136	44.4				
4 to 10	86	54	62.8				
> 10	22	13	59.1				
**SEASONS**							
Spring	315	149	47.3	Referent			
Summer	1128	538	47.7	0.741	0.545	1.009	0.057
Fall	396	135	34.1	1.005	0.705	1.433	0.979
Winter	342	90	26.3	0.652	0.445	0.955	0.028
**YEAR**							
2005	202	81	40.1	Referent			
2006	220	96	43.6	1.131	0.708	1.804	0.607
2007	224	94	42	1.265	0.798	2.004	0.318
2008	222	82	36.9	0.737	0.462	1.175	0.200
2009	158	55	34.8	0.662	0.396	1.108	0.117
2010	180	75	41.7	0.983	0.602	1.604	0.945
2011	177	71	40.1	1.022	0.625	1.672	0.930
2012	221	101	45.7	1.145	0.722	1.814	0.566
2013	155	58	37.4	0.938	0.562	1.567	0.808
2014	164	80	48.8	1.398	0.848	2.305	0.189
2015	140	59	42.1	1.225	0.734	2.044	0.437
2016	118	60	50.8	2.204	1.294	3.755	0.004
**AGE**							
Incomplete	603	418	69.3	Referent			
Complete	1578	494	31.3	1.148	0.776	1.696	0.490
**BCS**				1.849	1.605	2.130	< 0.001
1	223	28	12.6	Linear correlation			
2	907	329	36.3				
3	833	429	51.5				
4	218	126	57.8				

**DIAGNOSIS**							
Nestling	524	423	80.7	Referent			
Starvation	232	82	35.3	0.164	0.101	0.265	< 0.001
Head trauma	245	99	40.4	0.157	0.098	0.249	< 0.001
Multiple trauma	208	38	18.3	0.047	0.028	0.080	< 0.001
Wing fracture	649	160	24.7	0.079	0.051	0.123	< 0.001
Wing wound	36	10	27.8	0.085	0.036	0.201	< 0.001
Wing luxation	43	12	27.9	0.101	0.046	0.222	< 0.001
Leg trauma	70	25	35.7	0.130	0.071	0.239	< 0.001
Plumage trauma	59	37	62.7	0.388	0.200	0.752	0.005
Infectious or parasitic	31	18	58.1	0.267	0.116	0.616	0.002
Trunk trauma	20	1	5	0.010	0.001	0.077	< 0.001
Toxic	64	7	10.9	0.024	0.010	0.057	< 0.001
